# Predictors of Clostridium difficile infection after stoma reversal following TaTME surgery

**DOI:** 10.1007/s13304-023-01614-4

**Published:** 2023-08-04

**Authors:** Flavio Tirelli, Laura Lorenzon, Alberto Biondi, Lodovica Langellotti, Gloria Santoro, Annamaria Agnes, Roberto Pezzuto, Roberto Persiani, Domenico D’Ugo

**Affiliations:** General Surgery Unit, Fondazione Policlinico Universitario Agostino Gemelli IRCCS, Largo Francesco Vito 1, 00168 Rome, Italy

**Keywords:** Clostridium difficile, Stoma reversal, Ileostomy closure, Rectal cancer, TaTME

## Abstract

Background: The aim of this study was to determine the incidence of Clostridium Difficile infection (CDI) after stoma reversal in patients who underwent transanal Total Mesorectal Excision (TaTME) and to evaluate variables correlated with this post-operative infection. Methods: Patients who underwent stoma reversal surgery following TaTME for rectal cancer between 2015 and 2023 at a high-volume Institution, were retrospectively reviewed for the post-operative occurrence of diarrhea and in-hospital CDI (positive toxin in the stools). Patients were divided into the following subgroups according to the post-operative course: Group A-no clinical symptoms; Group B-mild diarrhea (< 10 evacuations/day); Group C-severe watery diarrhea (> 10 evacuations/day) with CDI negative; and Group D-severe watery diarrhea (> 10 evacuations/day) CDI positive. Clinical and laboratory data were analyzed for their correlation with CDI. A machine learning approach was used to determine predictors of diarrhea following stoma reversal. Results: A total of 126 patients were selected, of whom 79 were assessed as Group A, 16 Group B, 25 Group C and 6 (4.8%) Group D. Univariable analysis documented that delayed stoma reversal correlated with CDI (Group A mean interval 44.6 weeks vs. Group D 68.4 weeks, *p* 0.01). The machine learning analysis confirmed the delay in stoma closure as a probability factor of presenting diarrhea; also, diarrhea probability was 80.5% in males, 77.8% in patients who underwent neoadjuvant therapy, and 63.9% in patients who underwent adjuvant therapy. Conclusions: Stoma reversal surgery can result in moderate rate of in-hospital CDI. Time-to stoma reversal is a crucial variable significantly related with this adverse outcome.

**Supplementary Information:**

The online version contains supplementary material available at 10.1007/s13304-023-01614-4.

## Introduction

Clostridium Difficile Infection (CDI) is one of the most common healthcare-related infection [1].

Clostridium difficile is a Gram-positive, anaerobic, spore and toxin productor, that colonize gastrointestinal tract of 5–15% of the general population but can cause a serious disease in risk patients, including toxic megacolon, a rare but severe condition affected by substantial mortality rates [2].

Since the first description in the 1970s, the incidence, severity, and recurrence rate of CDI is increasing in literature [3].

CDI is most frequent after colon surgery compared to normal population (2.2% vs. 1%) and furthermore, the incidence in patient undergone colon surgery and stoma reversal is even higher (4%) when compared to other procedures [4]. Risk factors for CDI are old age, prolonged hospitalization, use of antibiotics, use of proton pump inhibitor, and immunosuppression [1].

The diagnosis of CDI requires the presence of watery diarrhea, and a positive stool test for its toxins, or endoscopic/histology findings demonstrating the presence of a pseudomembranous colitis [5].

Of note, the presence of diarrhea after stoma reversal is quite frequent, and it could be also related to a diversion colitis [6], thus the identification of this infection in patients who underwent stoma surgery could be misleading and difficult.

The primary outcome of this study was to determine the incidence of CDI after stoma reversal in the patients who underwent rectal cancer surgery by Transanal Total Mesorectal Excision (TaTME) approach in our Institution.

Secondary outcomes were to analyze the association between CDI and the clinical variables of the population to identify patients at higher risk to develop CDI after stoma reversal surgery.

## Methods

### Patients

This is a retrospective cohort study conducted at Fondazione Policlinico Universitario “A. Gemelli” IRCSS in Rome. Patients who underwent a TaTME for rectal cancer between April 2015 and April 2023 were retrieved from a prospectively maintained database, and their clinical medical records were reviewed and analyzed.

Patients were selected and included in this study if undergone TaTME with primary anastomosis and diverting stoma as a primary intervention and then performed stoma reversal surgery after being tested for the integrity of the anastomosis. Of note, the multidisciplinary management of all patients with rectal cancer treated at the Institution is discussed during weekly multidisciplinary team meetings. In brief, patients with cT3–cT4a N0 disease, or those staged cTN + , are usually scheduled for neoadjuvant chemoradiotherapy, consisting of 4 weeks of radiotherapy (total dose of 56 Gy) plus concomitant 5 fluoro-uracil, followed by delayed surgery after at least a 6-week interval.

The surgical technique has been standardized since its adoption, and the combined transanal/transabdominal procedure (Cecil approach) was introduced after the first eight sequential patients; all cases were performed by the same surgical team. Since 2015, the TaTME technique has been introduced at our unit and it has become the treatment of choice for patients with low and mid rectal cancers (1–6 cm and 7–11 cm from the anorectal junction, respectively).

Although a diverting stoma (eighter ileostomy or colostomy on the descending colon) is performed in most of the cases, the decision on whether to perform it or not is at surgeon’s discretion, based on clinical features (i.e., comorbidities, tumor height, neoadjuvant therapy, possible need for adjuvant therapy) and intraoperative findings (i.e., intraoperative anastomotic integrity tests positive for technical defects). After 6 weeks, patients usually perform a contrast-enhancement enema to check the integrity of the anastomosis and stoma reversal surgery is scheduled at that time or following the completion of adjuvant therapy if required. Ileostomy closure consist in a small bowel resection (resection of the stoma) and a latero-lateral anastomosis, whereas lateral colostomy closure is performed by direct suturing of the bowel defect. All patients subjected to stoma reversal receive a single dose of Cefazoline in prophylaxis.

### Diagnostic tests

As a general practice at our Institution, following stoma reversal, patients who present more than 10 diarrhea stools/day in the post-operative period, are tested for CDI, independently from other signs or symptoms of infection (i.e. abdominal pain, fever or laboratory values).

C. difficile test is usually performed on stool samples with the detection of C. difficile toxin A/B and glutamate dehydrogenase antigen (GDH).

If the stool sample results positive both for GDH and toxin A/B, CDI is confirmed; if GDH is positive but toxin A/B negative, a second level exam using acid nucleic amplification is required to confirm CDI.

### Outcomes of interest

The primary outcome of this study was to determine the incidence of CDI after stoma reversal. Secondary outcomes were to investigate the association between CDI and the main characteristics of the population, such as age, sex, BMI, Charlson index, albuminemia, neoadjuvant therapy, ASA score, adjuvant therapy and time to stoma reversal, to identify patients at higher risk of CDI and post-operative diarrhoea

### Statistics

Clinical variables were analyzed for their distribution (mean and standard deviation for normally distributed continuous variables; median and interquartile ranges for those non-normally distributed) and frequency and percentage distributions (categorical variables), and outliers were identified and excluded to preserve the goodness-of-fit of the data. Univariable parametric and not parametric analysis (Anova, Kruskal–Wallis and Fisher tests) were then performed to correlate variables with CDI. All tests were two-tailed and a *P*-value ≤ 0.05 was considered for the statistical significance.

Subsequently, a supervised machine learning approach was computed with different classification models, to differentiate and predict patients without diarrhea after stoma reversal vs others.

The data set was randomly partitioned, with 80% of the sample as training set and 20% of the sample as test-set. Due to the low sample size, an implementation with a 10-k fold cross-validation method was performed and based on this implementation. In order to increase accuracy of the analyses, a Random Forest (RF) classification model was designed, by the aggregation of many decision trees. Following, those models with acceptable values of accuracy, specificity and sensitivity were selected. Of note, the training set was double-checked for the parameters that controlled the complexity of the model, the relative error, the number of splits and trees to be generated. Finally, the entire model was checked for the control over the prediction using the confusion Matrix.

In order to improve prediction, we checked the absence of correlation among variables, and following we tested the dataset with the Naïve Bayes Algorithm, another type of supervised machine learning approach using a logic-based technique, based on Bayes’ Theorem that aims to compute the probability of the hypothesis given a prior knowledge, based on the following Bayesian’ s formula:

$${\text{P}}\left( {\text{A|B}} \right)\, = \,\frac{{{\text{P}}\left( {\text{B|A}} \right){\text{P}}\left( {\text{A}} \right)}}{{{\text{P}}\left( {\text{B}} \right)}},$$

where P(A): is the a priori probability (diarrhea Yes/No); P(B): represents the sum of all joint probabilities for each event j of A. This model assumes that the presence of one feature in a category is completely unrelated to the presence of all other features and the following parameters were evaluated: the Kernel Density Estimation (KDE: a nonparametric method used for pattern recognition and classification by density estimation in metric spaces to calculate the probability calculation of belonging to a group—diarrhea No/Yes); Bernoulli distribution; Bandwidth (the value of the bandwidth between the lowest and highest frequency value under curves).

All the analysis were performed using R software (https://www.r-project.org/), implementing with “CART”, “tree”, “randomForest” and “rpart” packages from CRAN Mirror (https://cran.r-project.org/mirrors.html) and naivebayes

## Results

### Patients and CDI

**Fig. 1:**
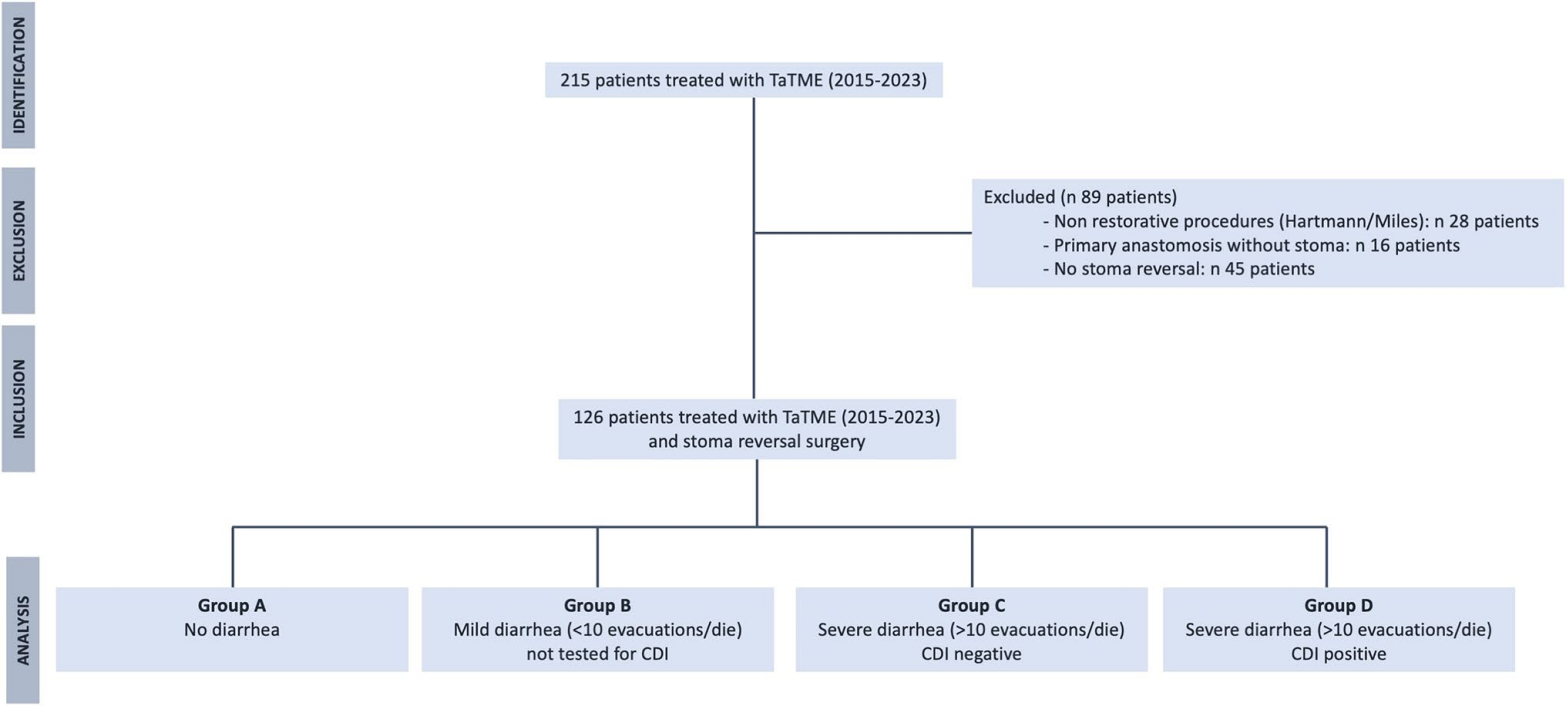
Study design and patients' selection. *TaTME*: transanal total mesorectal excision, *CDI* clostridium difficile infection

Figure 1 shows the study design and the selection flowchart of the case series. Out of the 215 consecutive patients who underwent rectal cancer resection using TaTME approach at the Institution, a total of 126 patients were selected (12 after colostomy closure and 114 after ileostomy reversal).

Eighty-nine were excluded since underwent nonrestorative procedures (Hartmann/Miles’ procedures; 28 patients), underwent surgery without diverting stoma (16 patients) or did not have stoma reversal yet (45 patients).

Demographical, clinical and post-operative features of the cohort are outlined in Table 1. The mean patient age at the surgical procedure was 66.6 ± 10.2. The female patients were 50 (39.7%) and the male patients were 76 (60.3%).

**Table 1:** Clinical features of 126 patients who underwent stoma reversal following TaTME for rectal cancer

Age, years	
Mean (SD)	66.6 (10.2)
Median	68
Range	61–75
Sex—*n* (%)	
Female	50 (39.7%)
Male	76 (60.3%)
BMI	
Mean (SD)	25.2 (4.1)
Median	24.9
Range	22.8–27.8
Missing	6 (4.8%)
Modified Charslon index -	
Mean (SD)	3.8 (2)
Median	3
Range	2–5
ASA score—*n* (%)	
ASA score 1	11 (8.7%)
ASA score 2	99 (78.6%)
ASA score 3	16 (12.7%)
Albumin, g/L	
Mean (SD)	40.4 (4.2)
Median	40
Range	38–42
Missing	2 (1.6%)
Interval between TaTME and stoma reversal, days	
Mean (SD)	232.2 (175.6)
Median	202.5
Range	13–1210
Neoadjuvant therapy before TaTME -n (%)	
Yes	91 (72.2%)
No	35 (27.8%)
Adjuvant therapy after TaTME and before stoma reversal -*n* (%)	
Yes	61 (48.4%)
No	65 (51.6%)
Sub-groups -*n* (%)	
Group A	79 (62.7%)
Group B	16 (12.7%)
Group C	25 (19.8%)
Group D	6 (4.8%)

The mean patient BMI was 25.2 ± 4.1, and the mean Charlson Index Score was 3.8 ± 2.0. Overall, 11 patients were classified as ASA 1 (8.7%), 99 patients as ASA 2 (78.6%) and 16 patients as ASA 3 (12.7%). The mean preoperative albuminemia was 40.4 g/L ± 4.2. Ninety-one patients underwent neoadjuvant therapy (72.2%), while 35 patients (27.8%) were treated with upfront surgery.

Sixty-one patients (48.4%) received adjuvant chemotherapy after TaTME and before stoma reversal surgery, instead 65 patients did not receive any adjuvant therapy or underwent adjuvant therapy after stoma closure.

The mean interval between the primary procedure and stoma reversal surgery was of 232.2 ± 175.6 days.

Thirty-one patients presented symptoms and were tested for CD, of which 6 patients were CDI positive.

**Fig. 2:**
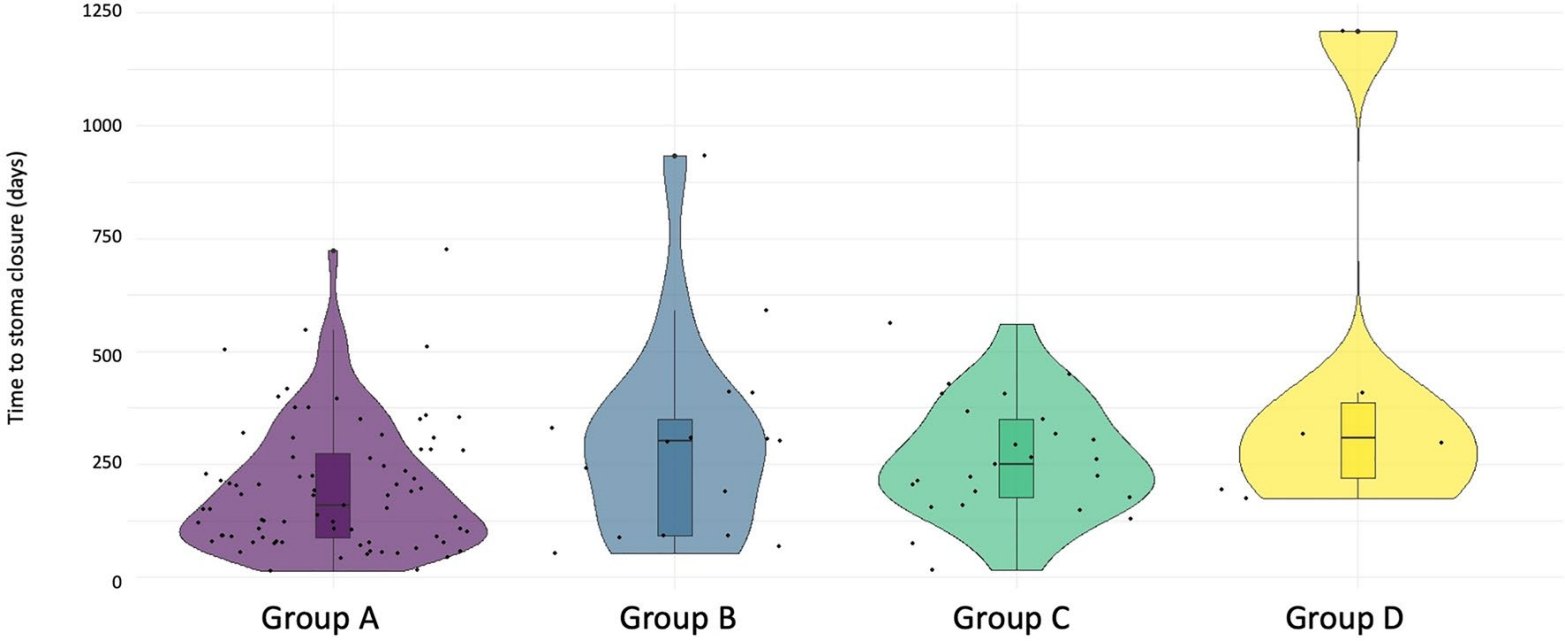
Mean time (days) between transanal Total Mesorectal Excision and stoma reversal in the 4 subgroups. Group A (79 patients; 62.7%)—no clinical symptoms; Group B (16 patients; 12.7%)—occurrence of mild post-operative diarrhea (< 10 evacuations/day) not tested for Clostridium Difficile Infection; Group C (25 patients; 19.8%)—presence of post-operative watery diarrhea (> 10 evacuations/day) but Clostridium Difficile Infection negative; and Group D (6 patients; 4.8%)—presence of post-operative watery diarrhea (> 10 evacuations/day) Clostridium Difficile Infection positive

On this basis, and according to the post-operative course, patients were divided into 4 subgroups: Group A (79 patients; 62.7%)—no clinical symptoms; Group B (16 patients; 12.7%)—occurrence of mild post-operative diarrhea (< 10 evacuations/day) not tested for CDI; Group C (25 patients; 19.8%)—presence of post-operative watery diarrhea (> 10 evacuations/day) but CDI negative; and Group D (6 patients; 4.8%)—presence of post-operative watery diarrhea (> 10 evacuations/day) CDI positive. After excluding 3 outliers for delayed stoma closure, the final the dataset consisted of 78 patients for Group A, 15 patients for Group B, 25 patients for Group C and 5 patients for Group D. All patients tested positive for CDI were treated with antibiotics according to international guidelines [7] and none developed complications or sequala. Univariable analysis documented that delayed stoma reversal correlated with CDI (Group A mean 44.6 days vs Group D 68.4 days, p 0.01), Table 2 and Fig. 2.

**Table 2:** Univariate analysis in the 4 subgroups

	Group A	Group B	Group C	Group D	*p*-value
Age, years (mean—SD)	66.5 ± 9.9	68.2 ± 13.3	65.1 ± 9.9	68.8 ± 9.2	0.76*
Sex—*n* (%)					
Female	38 (48.7%)	3 (20.0%)	7 (28.0%)	1 (20.0%)	0.06^$^
Male	40 (51–3%)	12 (80.0%)	18 (72.0%)	4 (80.0%)	
BMI (mean – SD)	24.9 ± 9.9	26.6 ± 13.3	25.2 ± 9.9	24.1 ± 9.2	0.51*
Modified Charslon Index (median – IQR)	3 (2—4)	3 (2—4)	5 (2—7)	3 (3—5)	0.47**
ASA score—*n* (%)					
ASA score 1	5 (6.4%)	3 (20.0%)	3 (12.0%)	0 (0.0%)	0.16^$^
ASA score 2	66 (84.6%)	9 (60.0%)	19 (76.0%)	3 (60.0%)	
ASA score 3	7 (9.0%)	3 (20.0%)	3 (12.0%)	2 (20.0%)	
Albumin (g/L.) (median—IQR)	40 (39–43)	40 (38–40)	40 (37–42)	40 (38–41)	0.72**
Interval to stoma reversal, weeks (mean-SD)	44.6 ± 29.4	57.9 ± 33.3	62.8 ± 26.2	68.4 ± 20.8	0.01*
Neoadjuvant therapy—*n* (%)					
Yes	55 (70.5%)	10 (66.7%)	18 (72.0%)	5 (100%)	0.53^$^
No	23 (29.5%)	5 (33.3%)	7 (28.0%)	0 (0.0)	
Adjuvant therapy—*n* (%)					
Yes	33 (37.5%)	6 (40.0%)	16 (64.0%)	4 (80.0%)	0.11^$^
No	45 (51.1%)	9 (60.0%)	9 (36.0%)	1 (20.0%)	

*Anova Test

**Kruskal–Wallis Test

^$^Fisher test

Bold values are for statistical significance

### Machine learning analysis

Based on these preliminary results, and due to the major number of categorical variables, we investigated data through a supervised machine learning approach considering patients without diarrhea condition after stoma closure *vs* others after excluding 3 outliers (total number of patients analysed: 123).

Therefore, Group A (patients without diarrhea) was considered as reference condition for splitting data.

According to this model, a BMI less than 24, was the main predictor of not having diarrhea. In the remaining population, the secondary predictor was an ASA score equal/greater than 2. Also, patients who had a delay (greater than the median value) in stoma closure had an increased probability to have symptoms. Finally, being female sex result to be a protective condition, Supplementary Fig. 1. Nevertheless, this model displayed weak accuracy (58%), sensitivity (60%) and specificity (25%).

**Fig. 3:**
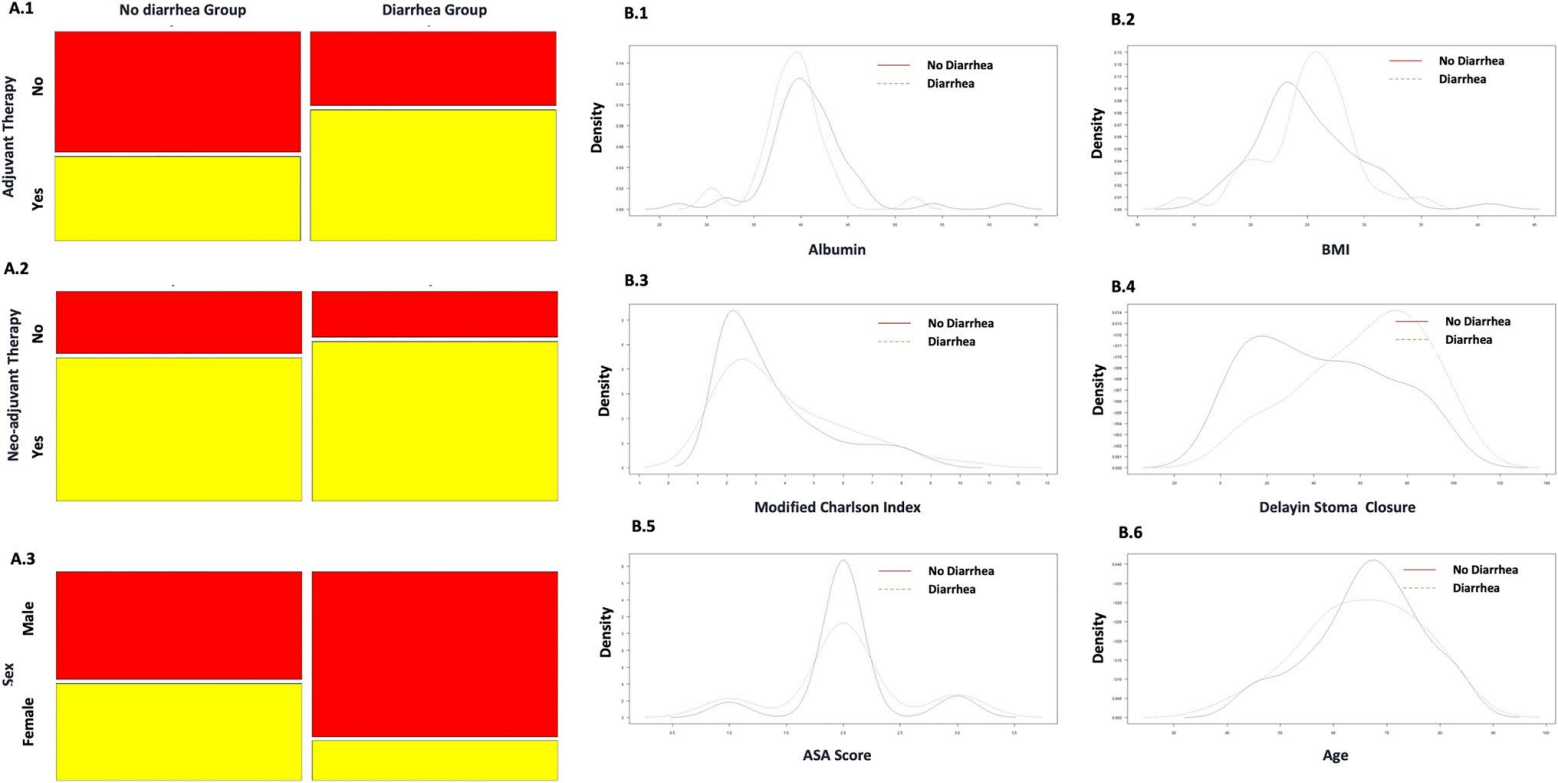
Graphical representation of the variables computed in the Naïve Bayes Algorithm analysis; Probabilities for the categorical variables and their distribution in the diarrhea and no-diarrhea groups: **A.1** Adjuvant therapy; **A.2** Neoadjuvant therapy; **A.3** Sex. Density estimation in continuous variables and their distribution in the diarrhea and no-diarrhea groups: **B.1** Albumin; **B.2** BMI; **B.3** Modified Charson Index; **B.4** Delay in stoma closure; **B.5** ASA score; **B.6** Age

On this basis, we tested the variables for any correlation (Supplementary Fig. 2) and given its absence, a Naïve Bayes Algorithm was used to show the probability of presenting or not presenting a post-operative diarrhea following stoma reversal. The graphical representation of each category is presented in Fig. 3, and the related probabilities in Table 3. As shown, the probability of presenting symptoms was 80.5% in the male population, 77.8% in patients who underwent neoadjuvant therapy before TaTME, and 63.9% in patients who underwent adjuvant therapy after the primary procedure and before stoma reversal. On the same extent, the probability of diarrhea was increased in those patients with increased co-morbidity, lower mean albumin values, and an increased delay of stoma closure.

**Table 3:** Naïve Bayes Algorithm

Variable (Distribution)	No Diarrea	Diarrhea
	Patients:78	Patients: 45
	Probability a priori: 63.4%	Probability a priori: 35.6%
Age at surgery, years (KDE)	Observations: 63	Observations: 36
	Bandwidth: 3.6	Bandwidth: 4.9
	Mean age: 63.5	Mean age: 61.5
Sex (Bernoulli)		
Male	52.3%	80.5%
Female	47.6%	19.4%
BMI (KDE)	Observations: 59	Observations: 35
	Bandwidth: 1.48	Bandwidth: 1.15
	Mean BMI:28.5	Mean BMI:24.5
Modified Charlson Index (KDE)	Observations: 63	Observations: 36
	Bandwidth: 0.58	Bandwidth: 0.93
	Mean Mod Ch. index.: 5.5	Mean Mod Ch. index: 6
ASA score (KDE)	Observations: 63	Observations: 36
	Bandwidth: 0.17	Bandwidth: 0.24
	Mean ASA score: 2	Mean ASA score: 2
Albumin (KDE)	Observations: 63	Observations: 35
	Bandwidth: 1.17	Bandwidth: 0.98
	Mean albumin: 44.5	Mean albumin: 41.0
Delay of stoma closure, weeks (KDE)	Observations: 63	Observations: 36
	Bandwidth: 11.3	Bandwidth: 11.6
	Mean delay: 49.5	Mean delay: 54.0
Neoadjuvant therapy (Bernoulli)		
No	30.2%	22.3%
Yes	69.8%	77.8%
Adjuvant therapy (Bernoulli)		
No	58.7%	36.1%
Yes	41.3%	63.9%

*KDE* kernel density estimation, *Mod Ch. Index* modified Charlson index

This model has a mis-classification percentage of about 20% with, and the accuracy on training set was about 80%. On the other hand, the prediction on test-set on patients without diarrhea had a 37% of mis-classification and a total accuracy of 63%.

## Discussion

Clostridium difficile is a Gram-positive spore-forming bacterium that produces two toxins, A and B, that cause disease. The organism is oro-fecal transmitted symptoms range from mild diarrhea to life-threatening fulminant colitis. It is among the most common health-related gastrointestinal infections [1, 8].

Asymptomatic colonized individuals with no clinical signs can act as an infection reservoir and transmit it to others. The prevalence of asymptomatic colonization among healthy adults with no prior risk factors for CDI is between 0 and 15% [1].

CDI is most frequent after colon surgery (2.2% vs. 1%) compared to normal population; furthermore, the incidence in patient undergone colon surgery with diverting stoma is even higher (4%) when compared to other surgeries [4].

A recent systematic review published in 2019, showed that the overall incidence of CDI is about 0,22% of hospitalized patient [9].

Risk factors for CDI are old age, prolonged hospital staying, use of antibiotics, use of proton pumps inhibitor, immunodepression, inflammatory bowel disease and surgery [1, 8].

Gastrointestinal microbiota plays a crucial role in protecting the intestines by providing resistance to colonization and infection by pathogenic organisms. Disruption of the normal gut flora allows *C*. difficile to proliferate and produce toxins [1, 10].

An association between CDI and antimicrobial treatment > 10 days has also been demonstrated, but also very limited exposure, such as single-dose surgical antibiotic prophylaxis, can increase patients’ risk for both C. difficile colonization and infection [1].

Antibiotics associated with the highest risk of CDI are clindamycin, cephalosporins, and fluroquinolones [1], as well as the use of proton pump inhibitors [11].

Colorectal surgery is a documented risk factor for CDI. A retrospective analysis of 84,648 patients undergoing colorectal surgery between 2008 and 2012 in USA showed that CDI occurred in 1,5%. The strongest predictors of CDI were emergency procedure, inflammatory bowel disease, and severity of illness score. CDI was associated with a higher rate of complications, intensive care unit (ICU) admission, longer preoperative inpatient stay, 30-day readmission rate, and death within 30 days compared to non-CDI patients [12].

Another retrospective colectomy database review of 2015 demonstrated that stoma reversal, smoking, steroids, and disseminated cancer were associated with CDI in the 30-day post-operative period [13].

A de-functioning ileostomy is performed during colorectal surgery in order to divert the intestinal content and protect distal anastomosis. It is most frequent after rectal cancer surgery, because this type of anastomosis has an increased risk of leakage, due to the site and to the fact that patients are often pre-treated with chemotherapy and radiotherapy.

Temporary stoma is usually closed a few months after primary surgery, after confirming with radiological study the integrity of the distal anastomosis. Stoma reversal surgery can be early (within 90 days after primary surgery) or late (after 90 days). Generally, patients undergoing adjuvant chemotherapy are candidate to late stoma reversal. Elective closure of ileostomy is usually considered a low-risk operation with a mortality rate of 0.4%.

Randall et al. [14] in 2009 analyzed the incidence of CDI in patient underwent colorectal surgery, studying a cohort of patient admitted for colorectal surgery and found out positive for CD after surgery. In their study, the incidence of CDI following ileostomy closure was 4.2%, twice that observed for right hemicolectomy and four times that for anterior resection. Patients undergoing ileostomy closure may be at greater risk than those having other procedures for several reasons. First, they have undergone a previous surgical procedure with the resulting hospital stay and antibiotic usage. Secondly, studies in animal models have shown that the defunctioned colon undergoes mucosal and muscular atrophy [15]. It is conceivable that parallel physiological and microbiological changes in the small bowel and defunctioned colon may result in infective diarrhea when the colon is brought back into circuit after stoma closure.

Another systematic review of 2017, including 11 papers [16], established the incidence of Clostridium difficile infection (CDI) of 1.8% after ileostomy reversal.

Kim et al. [17] underlined that Clostridium difficile infection is reported to be frequent in patients who receive ileostomy closure for rectal cancer. In his paper, a total of 1270 patients were included, and 208 patients were tested for CDI owing to colitis-related symptoms. The incidence of CDI was 3.6 per cent (46 patients). Furthermore, while previous studies have reported comorbidities as DM, heart conditions, chronic renal to be risk factors for CDI, this paper revealed that only adjuvant chemotherapy and anastomosis leakage (and consequently a prolonged exposure to antibiotics) are independent risk factors for CDI.

With this research, we documented that a delayed stoma closure is the main variable correlated with CDI after stoma reversal following TaTME for rectal cancer. The machine learning approach documented also the probability presenting with diarrhea symptoms for each variable evaluated and highlighted the clinical features of patients presenting this adverse post-operative course (male sex, increased co-morbidity, lower mean albumin values, increased stoma delay closure, use of neoadjuvant and adjuvant treatments).

Limits of this research regards the relatively small population, and the absence of external validation of our findings, however, a major strength relays in the case series which is homogeneous for treatments and protocols used and belong to a high-volume Institution for rectal cancer surgery.

## Conclusions

Stoma reversal surgery can result in moderate rate of in-hospital CDI. Time-to stoma reversal is a crucial variable correlated with this adverse outcome.

## Supplementary Information

Below is the link to the electronic supplementary material.

Supplementary file1 (JPG 171 KB) Supplementary Figure 1. Random Forest analysis to predict absence of diarrhea following stoma reversal using clinical variables.

Supplementary file2 (JPEG 364 KB) Supplementary Figure 2. Correlation test in clinical and laboratory variables.

## Data Availability

Research data supporting this publication are available from the corresponding author on reasonable request.
