# Supplementary figures and images for: Predictors of Clostridium difficile infection after stoma reversal following TaTME surgery
Source: Updates Surg. 2023 Aug 4;75(6):1589–96. doi: 10.1007/s13304-023-01614-4 (PMC10435656; doi:10.1007/s13304-023-01614-4)

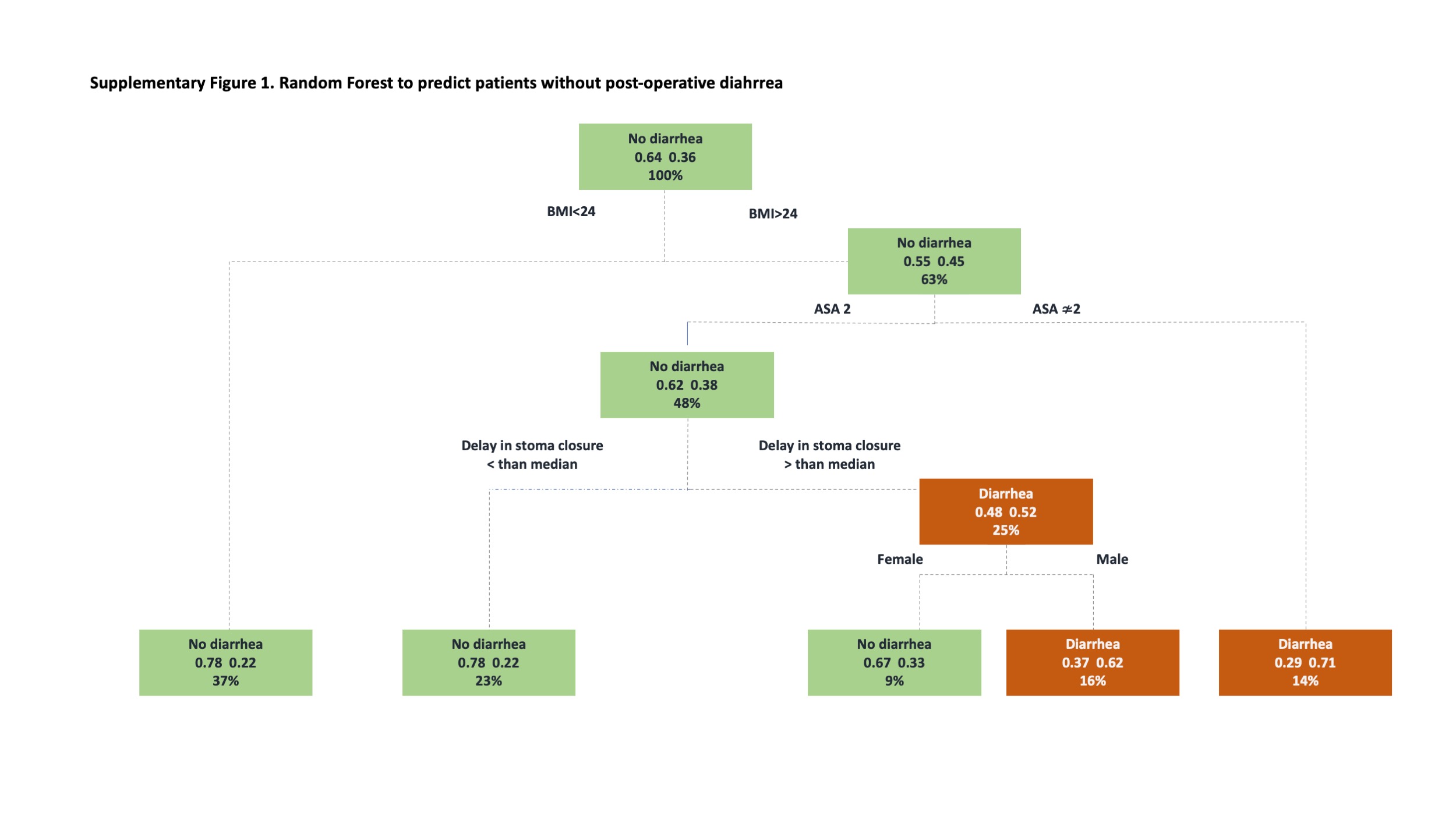

Supplement: Supplementary file 1 — Supplementary file1 (JPG 171 KB) Supplementary Figure 1. Random Forest analysis to predict absence of diarrhea following stoma reversal using clinical variables. [file 13304_2023_1614_MOESM1_ESM.jpg]

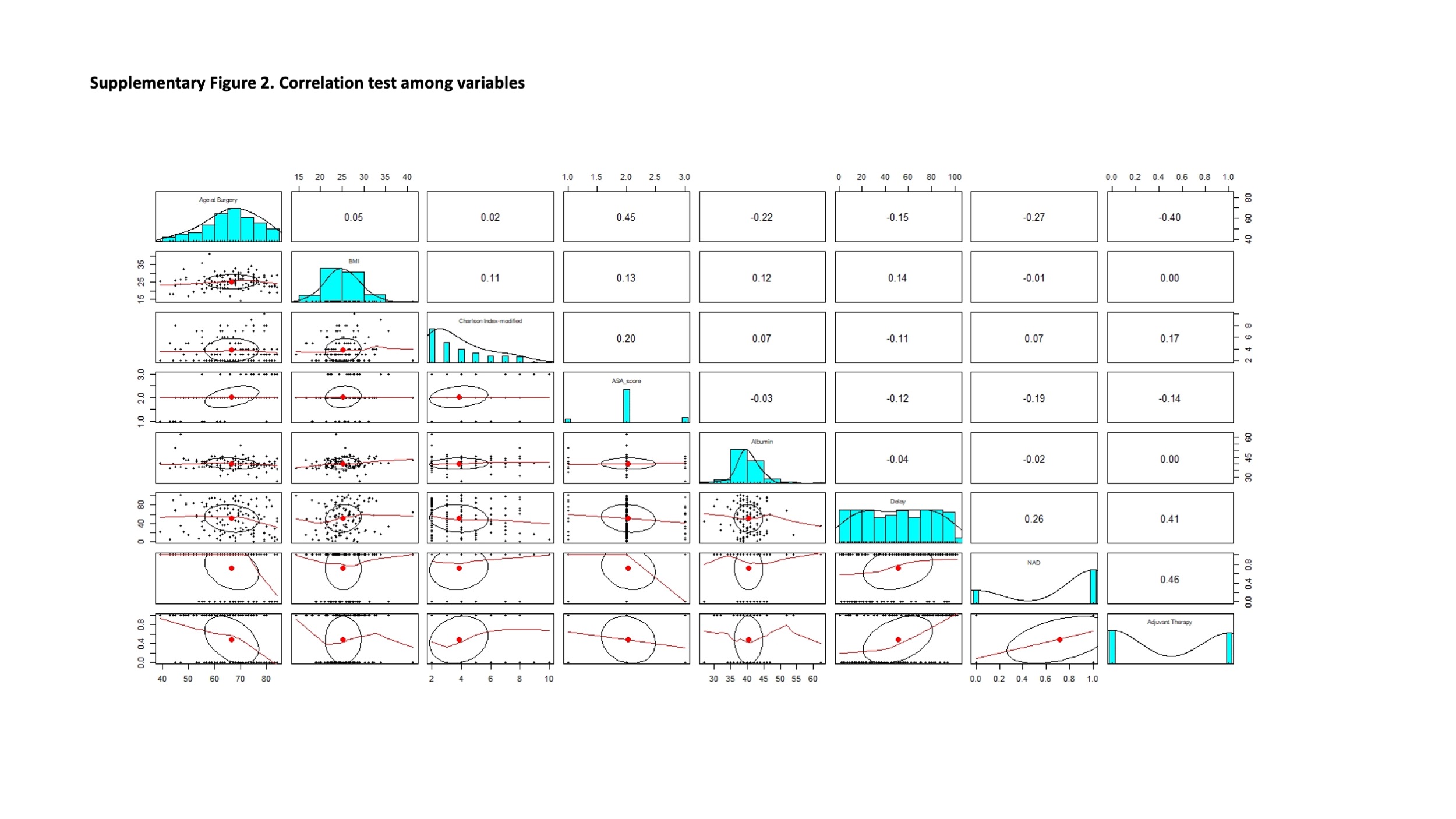

Supplement: Supplementary file 2 — Supplementary file2 (JPEG 364 KB) Supplementary Figure 2. Correlation test in clinical and laboratory variables. [file 13304_2023_1614_MOESM2_ESM.jpg]
